# New Challenges in the Study of Liver Diseases: From Molecular Pathogenesis to Therapeutic Approaches (3rd Edition)

**DOI:** 10.3390/biomedicines13122879

**Published:** 2025-11-26

**Authors:** Paola Dongiovanni, Marica Meroni

**Affiliations:** Medicine and Metabolic Diseases, Fondazione IRCCS Cà Granda, Ospedale Maggiore Policlinico, 20122 Milan, Italy; paola.dongiovanni@policlinico.mi.it

## 1. Introduction

The liver is defined as the central metabolic hub of the body, harmonizing nutritional, hormonal, and catabolic stimuli to preserve systemic homeostasis [1,2,3]. Due to its peculiar structure and anatomical position, being situated between the digestive tract and systemic circulation, it is continuously exposed to dietary, microbial, toxic, hormonal, and immune-derived insults, thus preserving communication with the entire digestive system [4]. Therefore, its physiological function allows the detection and processing of a broad array of metabolic, gut-derived, and immunological signals which make this organ vulnerable to several injuries, including metabolic overload, toxins, infections, and immune alterations [5,6]. Deviations from hepatic physiology are particularly harmful; hence, the increasing number of affected patients represents a global health concern [7].

Liver diseases constitute the most heterogeneous and pressing challenge in medicine, encompassing an umbrella of hepatic disorders ranging from metabolic-dysfunction-associated fatty liver disease (MASLD), cholestatic injuries, and viral or toxic hepatitis to alcohol-related cirrhosis, hepatocellular carcinoma (HCC), and fulminant liver failure [8]. These conditions may share complex pathological substrates shaped by the interaction between genetics, epigenetics, metabolic, and environmental risk factors [9]. Furthermore, hepatic failure is often associated with an increased risk of developing extrahepatic comorbidities, such as cardiovascular, renal, intestinal, systemic, and cerebral complications [10].

Although our understanding of their etiopathogenic basis has advanced considerably, many liver diseases remain underdiagnosed, and therapeutic options are still limited. Furthermore, liver biopsy continues to be the diagnostic gold-standard procedure for treating many of them, indicating the urgent need to identify reliable non-invasive biomarkers to guide clinical management [11]. In recent years, the focus has shifted toward integrating molecular biomarkers, novel experimental models, and targeted therapeutic strategies, delving into molecular mechanisms and strategies aimed at bridging bench discoveries and clinical interventions. Despite advances in this direction, therapeutic options remain inadequate, and diagnostic strategies often fail to identify the diseases early or arrive at a prognosis. Hence, the translation of molecular discovery into effective diagnostic and therapeutic strategies remains incomplete.

This Special Issue, New Challenges in the Study of Liver Diseases: From Molecular Pathogenesis to Therapeutic Approaches (3rd Edition), gathers diverse contributions that highlight both mechanistic insights and translational approaches. The seven articles presented here collectively illustrate how the field is tackling the demand for novel biomarkers, the systemic effects of liver injury, immune alterations, and possible limitations of antioxidant therapies. The purpose of this Editorial is to provide a brief overview of recent developments in this multifaceted field and encourage the readers to explore these contributions.

## 2. An Overview of the Published Articles

The first contribution in this Special Issue is the study by Mobasher et al. [12], who explored the role of the long non-coding RNA growth-arrest-specific 5 (lncRNA GAS5), along with miR-29a-3p and neurogenic locus notch homolog protein 2 (NOTCH2), in non-alcoholic fatty liver disease (NAFLD). The authors demonstrated that lncRNA GAS5 was highly expressed in NAFLD patients and even more so in those affected by cirrhosis. In particular, they concluded that this long non-coding molecule may have diagnostic utility as a noninvasive biomarker, constituting a molecular tool that can better stratify NAFLD patients and potentially improve detection strategies. Beyond its diagnostic significance, GAS5 participates in a broader network of non-coding RNAs that modulate lipid metabolism, apoptosis, and inflammation—hallmarks of NAFLD pathogenesis [13]. In line with these results, other studies have demonstrated its regulatory role in the onset of fibrosis in various organs [14,15].

The growing epidemic of NAFLD, recently redefined as metabolic-dysfunction-associated steatotic liver disease (MASLD) [16], compounds the urgent need to discover trustworthy biomarkers capable of detecting the onset of disease early and predicting its progression up to end-stage conditions. This study provides evidence supporting the possibility of using non-coding molecules as predictors of disease course and links altered transcriptomic profiles and regulatory networks to MASLD pathogenesis, indicating new molecular targets for intervention.

Along with identifying novel disease tracers, making therapeutic advances remains a pressing mission. In this regard, Lohvinenko et al. [17] explored the hepatoprotective potential of an S-substituted pteridine in a rat model of CCl_4_-induced acute hepatitis. This compound demonstrated antioxidant and cytoprotective properties superior to those of the reference drug Thiotriazoline, pointing to new opportunities for drug development regarding synthetic antioxidants with thiol functionality. While MASLD remains the most prevalent liver disease globally [18], toxic liver injuries remain a frequent cause of acute hepatic decompensation, and effective therapies are still lacking. Together with emerging data on endogenous redox systems such as glutathione, these findings reinforce the central role of redox balance in hepatic protection, offering a novel therapeutic path.

Oxidative stress is a common denominator across multiple liver diseases [19,20]; accordingly, two contributions in this Special Issue evaluate antioxidant strategies for counteracting their progression towards end stages. Nguyen and colleagues [21] reviewed the therapeutic potential of glutathione supplementation in mitigating oxidative and endoplasmic reticulum stress occurring during MASLD. As the body’s most abundant antioxidant, glutathione seems to possess the ability to improve mitochondrial redox homeostasis and insulin sensitivity, thus ameliorating the oxidative damage that fuels the progression from simple steatosis to steatohepatitis and cirrhosis. The authors highlight encouraging preclinical and clinical evidence in this regard but also stress the need for standardized dosing regimens and larger randomized trials before glutathione can be recommended for use in routine care.

Complementing this review, Orban et al. [22] presented a meta-analysis of 266 randomized controlled trials (RCTs) on intravenous N-acetylcysteine (NAC) in *non-paracetamol*-induced acute liver failure. In contrast to its well-established role in acetaminophen’s toxicity, NAC led to no significant improvements in survival rates or hospitalization outcomes in other etiologies. This negative finding is just as important as a positive finding. Indeed, these results may temper the enthusiasm for extending antioxidant therapies indiscriminately and highlight the necessity of adopting a disease-specific mechanistic rationale when designing treatment strategies.

Several hepatic disorders may devolve into fibrosis, cirrhosis, HCC, including MASLD, viral hepatitis, and the TGF-B signaling cascade plays a central role in this process [23]. In the context of viral hepatitis, Weber and colleagues [24] investigated the interplay between fibrosis and HCC risk by focusing on the bone morphogenetic protein and activin membrane-bound inhibitor (BAMBI). The latter is a pseudoreceptor that acts as an endogenous inhibitor of TGF-B signaling activation [25]. The authors demonstrated that BAMBI hepatic levels decline in hepatocytes during chronic hepatitis-C-related fibrogenesis. However, its expression was not clearly linked to the development of HCC, and its downregulation was mediated by the cellular context and crosstalk with other pathways. Thus, this work refines our understanding of TGF-B regulation in chronic viral hepatitis and disentangles the development of fibrosis from carcinogenesis. Furthermore, this evidence corroborates the importance of considering disease etiology before making generalized conclusions about a therapeutic target.

Along with metabolic and viral etiologies, alcohol-induced liver disease (ALD) is one of the leading causes of hepatic cirrhosis worldwide [26]. In 281 male patients with alcoholic cirrhosis, Legaz and colleagues [27] examined alterations in innate immunity, particularly in killer-cell immunoglobulin-like receptor (KIR) signaling, and their role in influencing susceptibility to viral infections, including viral hepatitis C (HCV), hepatitis B (HBV), and cytomegalovirus (CMV). The authors concluded that alcohol not only seems to exert a direct hepatotoxic effect but also represents an indirect modulator of immune surveillance, offering a fertile soil for viral infections due to the impairment of antiviral defenses. In detail, the screening of KIR genes and HLA-C genotypes allowed the authors to reveal that KIR2DL2/C2C2 genetic profiles may primarily act in determining patients’ vulnerability to viral infections. This dual burden complicates the management of ALD patients. Thus, these observations pave the way for tailored immunomodulatory approaches to ALD cirrhosis, enhancing the activation of the cytotoxic activity of the NK cells of these patients.

Liver diseases have been broadly correlated with several extra-hepatic comorbidities, including cardiovascular disorders [28,29]. In this regard, using an experimental rodent model, Billing et al. [30] found that acute cholestasis may induce coronary microvascular dysfunction by increasing levels of bile acids and transaminases, reducing cardiac output, and impairing coronary flow reserves. The main findings corroborated the notion that hepatic injuries may directly affect cardiac performance, revealing a direct liver–heart communication likely mediated by inflammatory and endothelial factors. This study identifies microcirculatory impairment as the link between liver damage and cardiac stress and further stresses the need to consider patients affected by liver diseases from a multi-systemic point of view.

These articles align with the emerging concept of *precision medicine*, which integrates molecular diagnostics, immune profiling, and cross-organ interplay into personalized diagnostic paths and therapeutic frameworks, which necessitate multidisciplinary interventions. The convergence of omics technologies, advanced imaging, and computational modeling via machine learning approaches will further refine our understanding of liver diseases as dynamic network disorders rather than isolated pathologies [31].

## 3. Conclusions

These studies capture the ongoing transformation of liver disease research from descriptive pathology to mechanism-based and translational science. From the search for circulating biomarkers and novel pharmacological agents to unravelling the complex links between fibrosis, immunity, systemic dysfunction, and oxidative stress, the field is moving towards a more integrative vision of liver diseases. The heterogeneity of these conditions implies that no single biomarker or therapy will suffice; instead, precision medicine approaches, tailoring interventions to molecular profiles, immune status, and comorbid organ systems, will be increasingly required. In addition, we require multidisciplinary collaborations that bridge molecular biology, pharmacology, immunology, and clinical medicine to move forward in this field, making the management of the affected patients a concrete holistic matter [10,32].

In this context, multiomics approaches integrating single-cell resolution, spatial reconstruction, and artificial-intelligence-based solutions will boost both clinical practices and scientific discoveries. Therefore, progress in our understanding of liver diseases will depend on the continued convergence of basic science, translational research, and rigorous clinical evaluation in line with the concept of ‘*From bench to bedside and back again*’. By bridging these domains, the community can move closer to the ultimate goal of effective, personalized, and preventive care for patients, helping to tackle the burden of liver disease worldwide [33].
